# Laser-Induced Solid-Phase UV Fluorescence Spectroscopy for Rapid Detection of Polycyclic Aromatic Hydrocarbons in the Land Snail Bioindicator, *Cantareus aspersus*

**DOI:** 10.3390/bios15070450

**Published:** 2025-07-14

**Authors:** Maxime Louzon, Thomas Bertoncini, Noah Casañas, Yves Perrette, Gaël Plassart, Marine Quiers, Tanguy Wallet, Mohamed Kamel, Lotfi Aleya

**Affiliations:** 1 ENVISOL, 2 rue Hector Berlioz, 38110 La Tour du Pin, France; thomas.bertoncini38@gmail.com (T.B.); n.casanas@envisol.fr (N.C.); g.plassart@envisol.fr (G.P.); marine.quiers@gmail.com (M.Q.); t.wallet@envisol.fr (T.W.); 2 EDYTEM (Environment, Dynamic and Mountain Territories), CNRS, Savoie-Mont Blanc University, 5 Boulevard de la Mer Caspienne, 73370 Le Bourget-du-Lac, France; yves.perrette@univ-smb.fr; 3 Department of Medicine and Infectious Diseases, Faculty of Veterinary Medicine, Cairo University, Giza 11221, Egypt; 4 Laboratoire de Chrono-Environnement, Université de Bourgogne Franche-Comté, UMR CNRS 6249, La Bouloie, 25030 Besançon, France; lotfi.aleya@univ-fcomte.fr

**Keywords:** bioindicators, bioaccumulation, transfers, solid-phase spectroscopy, laser-induced UV spectroscopy, snails

## Abstract

In ecotoxicological risk assessment, current methods for measuring the transfer and bioavailability of organic pollutants like polycyclic aromatic hydrocarbons (PAHs) in bioindicators are often destructive and environmentally unfriendly. These limitations are especially problematic when only small amounts of biological material are available. Here, we present a novel, high-throughput method combining laser-induced UV fluorescence spectroscopy (UV-LIF) and solid-phase spectroscopy (SPS) for rapid, in situ quantification of PAHs in land snails—a key bioindicator species. Using dual excitation wavelengths (266 nm and 355 nm), our method reliably detected pyrene and fluoranthene in snails exposed to varying concentrations, demonstrating clear dose-responses and inter-individual differences in bioaccumulation. The analysis time per sample was under four minutes. This approach allows simultaneous measurement of internal contaminant levels and health biomarkers in individual organisms and aligns with green chemistry principles. These findings establish a new, scalable tool for routine assessment of PAH transfer and bioavailability in diverse ecosystems.

## 1. Introduction

In environmental toxicology, accurately assessing the bioavailability of contaminants within ecosystems is critical for effective risk evaluation and management. To this end, bioindicators serve as essential tools by providing direct evidence of contaminant uptake and accumulation in organisms, reflecting the biological impact of pollutants in both aquatic and terrestrial environments [1,2]. Specifically, in soil ecotoxicology, bioindicators are integral to the Weight of Evidence (WoE) framework, which integrates chemical, toxicological, and ecological lines of evidence to reduce uncertainties and improve the reliability of site-specific risk assessments [3,4]. Thus, bioavailability assessment using bioindicators bridges the gap between contaminant presence and observed biological effects, strengthening the conclusions. This is particularly effective when used in the TRIAD approach, which links contaminant concentrations in soil with biological and ecological disturbances observed under laboratory and field conditions (ISO 19204:2017 [4]). More precisely, in site-specific ecological risk assessment, the TRIAD approach combines three complementary lines of evidence: (i) chemical analyses to characterize contaminant levels and transfers to organisms, (ii) ecotoxicological bioassays and biomarkers to evaluate potential toxic effects on model organisms, and (iii) ecological field surveys to detect disturbances in local communities or functions. By integrating these components, the methodology provides a WoE framework that accounts for both exposure and ecotoxicological relevance. This approach allows for a more realistic and operational evaluation of risks in complex environmental settings. Among persistent organic pollutants, polycyclic aromatic hydrocarbons (PAHs) are particularly concerning due to their widespread distribution and propensity to bioaccumulate in terrestrial organisms, such as land snails (*Cantareus aspersus*) [5]. However, conventional chemical methods for assessing PAH bioavailability often face limitations related to extraction efficiency and interpretative challenges, making biological approaches valuable complements [6]. In this context, terrestrial snails, positioned at the soil-air-plant interface, offer a practical and standardized means of assessing PAH bioavailability through both in situ and ex situ exposure scenarios [2,7]. Moreover, advances in analytical techniques, such as improved extraction protocols, such as QuEChERS, have enhanced the reliability of snail-based bioindicators in environmental monitoring. In addition, the establishment of threshold values for risk indices, such as GSET and GERITOXE, has further strengthened their application in this field [5,8].

The quantification of PAHs in bioindicator organisms predominantly relies on gas chromatography coupled with tandem mass spectrometry (GC-MS/MS) following extraction methods such as QuEChERS [2,5]. Although effective and widely adopted, this approach has several drawbacks. It requires substantial sample destruction, consumes high amounts of reagents and energy, generates considerable plastic and laboratory waste, and often limits the possibility of performing multi-contaminant or biomarker analyses from limited biological material [9,10]. These constraints underscore the urgent need for alternative methodologies that are non-destructive, cost-effective, and environmentally sustainable. These alternatives align with broader scientific efforts to reduce animal testing, including for invertebrates, and to optimize sample use in ecotoxicological research [11].

In response to these challenges, laser-induced UV fluorescence (UV-LIF) spectroscopy in the solid phase has emerged as a promising analytical technique. Characterized by rapid analysis, minimal sample preparation, non-destructiveness, eco-friendliness, and low operational costs, UV-LIF offers distinct advantages over conventional methods [12,13,14,15]. Despite its successful application in petroleum hydrocarbon analysis [13,16], UV-LIF has not yet been explored for quantifying PAHs bioaccumulated in terrestrial bioindicators.

Therefore, this study aims to evaluate the feasibility of solid-phase UV-LIF spectroscopy for detecting PAHs transferred to the land snail *C. aspersus*. To achieve this goal, pyrene and fluoranthene were selected as model compounds because of their well-characterized fluorescence properties (fluoranthene with a peak near 460 nm and pyrene exhibiting peaks at approximately 370 and 395 nm) and their environmental relevance. These PAHs are particularly significant given their physicochemical properties, including similar log Kow values (~4.90 and ~4.88, respectively), contributing to their persistence and bioaccumulation potential. Furthermore, their widespread presence in contaminated environments, such as the Seine River Basin, where they represent 15–46% of the total detected PAHs, reinforces their selection as target analytes for ecotoxicological monitoring [17,18].

By developing a minimally invasive method that preserves biological samples for subsequent analyses, including metal quantification or biomarker measurements incompatible with standard PAH extraction techniques, this approach seeks to provide a valuable complementary tool for ecotoxicological assessments. Ultimately, integrating UV-LIF spectroscopy into bioindicator analysis could enable the rapid screening of PAH bioavailability while supporting sustainable laboratory practices. This advancement will contribute to improving ecotoxicological risk assessments and rationalizing animal use in environmental monitoring programs.

## 2. Materials and Methods

### 2.1. Biological Material (Snails)

Young adult *C. aspersus* snails (8 g ± 1) were obtained from a certified snail farm (17187, Guitinières, France) and verified to be free of PAH contamination. Prior to exposure, snails were maintained for one month under controlled laboratory conditions, including a temperature of 20 °C, an 18 h light/6 h dark photoperiod, and an average relative humidity of 60%. During this acclimation period, the snails were fed uncontaminated Helinove food (SARL Le Gastéropote, 85500, Saint Paul en Pareds, France), consistent with ISO 24032:2021 standard [7].

### 2.2. Chemicals

The chemicals used in this study included pyrene (CAS 129-00-0), fluoranthene (CAS 206-44-0), and ethanol (CAS 64-17-5), all purchased from Alfa Aesar (Ward Hill, MA, USA), with purities of 98%, 98%, and 96%, respectively. These reagents were used to spike the snail food for exposure experiments.

### 2.3. Exposure Procedure

**Figure 1:**
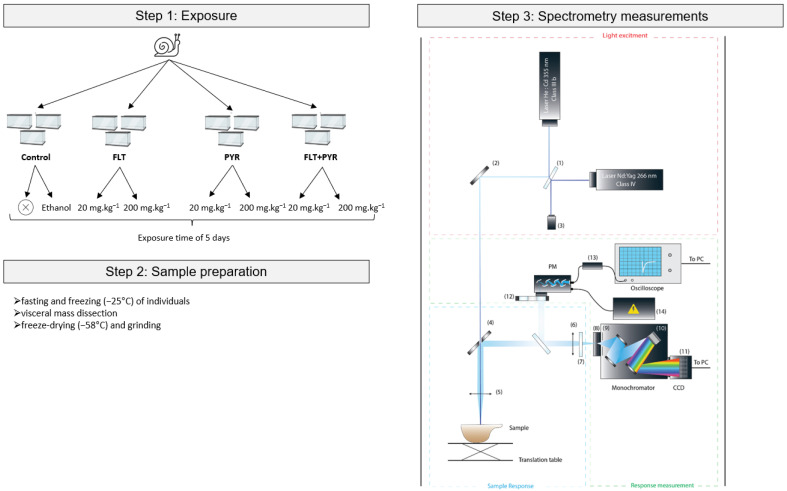
Experimental Workflow: Exposure, Sample Preparation, and Spectrometry Measurements (the control in step 1 refers to the sample without any PAH contamination). The negative control includes two sub-controls: one without solvents and one with solvents. The solvent control (ethanol) represents the sample in which the PAH solvent vector is added, but no PAHs are introduced, serving to assess the potential impact of the solvent. Each exposure modality was repeated in triplicate, with six snails placed in each replicate. For step 3, the different parts of the equipment are (1) beamsplitter, (2) mirror, (3) trigger (photodiode), (4) pierced mirror, (5) and (6) lens 1f, (7) high-pass filter, (8) slot, (9) shutter, (10) double motorized network (300 grt/mm–1200 grt/mm), (11) CCD 256 × 2048 px, (12) filter wheel, (13) signal transformer A > V, and (14) photomultiplier (PM) power supply.

The overall experimental workflow is outlined in Figure 1. To evaluate the bioaccumulation of PAHs, snails were orally exposed ad libitum to Helinove food spiked with pyrene and fluoranthene at two concentrations: 20 mg·kg^−1^ (low dose) and 200 mg·kg^−1^ (high dose). These compounds were tested both individually (PYR 20, PYR 200, FLT 20, FLT 200) and in combination (PYR+FLT 20, PYR+FLT 200) (Table 1). Prior to spiking, PAHs were dissolved in ethanol and applied to the Helinove food, which was then freeze-dried at −56 °C to ensure complete evaporation of the solvent.

To control for the potential effects of the spiking solvent, two negative controls were included: unspiked Helinove and Helinove spiked with ethanol alone (solvent control). For each experimental condition, eighteen snails were housed in ExoTerra faunarium boxes divided into three replicates of six individuals each. The exposure lasted five days under the same controlled environmental conditions as acclimation. Food was replaced every 36 h, and feces were removed regularly to prevent re-ingestion of metabolized PAHs.

Following exposure, the snails underwent a two-day fasting phase to clear their digestive systems of residual food. Subsequently, they were weighed and immediately frozen at −58 °C. Two individuals per replicate (six per condition) were randomly selected for analysis to provide sufficient sample material, while unselected specimens were discarded. The visceral mass was dissected from each snail after thawing, freeze-dried at −58 °C for 48 h, and manually ground using a porcelain mortar and pestle. This organ was chosen because of its known role as a primary site of PAH bioaccumulation.

### 2.4. Spectrofluorescence Measurements

Fluorescence analysis of the ground visceral mass samples was conducted using laser-induced ultraviolet fluorescence (UV-LIF) spectroscopy with a custom instrument, as described by Garagnon et al. 2023 [19] (Figure 1). This setup includes two pulsed Nd:YAG laser excitation sources operating at wavelengths of 266 nm (>0.3 µJ per pulse) and 355 nm (>1 µJ per pulse), both running at a frequency of 10 kHz.

The optical path directs the laser beams through a 50:50 beam splitter and a perforated mirror onto the sample surface. The fluorescence emitted by the sample is collected via a lens into a monochromator (Horiba Jobin Yvon MicroHR Series, Kyoto, Japan) equipped with a 300 grooves/mm diffraction grating blazed at 550 nm. Emission spectra covering the range from 283 nm to 957 nm were recorded using a back-illuminated CCD detector (JY-Syncerity, Horiba, Kyoto, Japan), which provides high sensitivity in the ultraviolet region.

To capture the spatial variability within the samples, a surface area of 5 × 5 mm was scanned using incremental steps of 0.5 mm, acquiring approximately 110 spectra per sample. Each spectrum was integrated over 100 ms. All measurements were performed in complete darkness within a climate-controlled laboratory environment maintained at 18 °C to ensure consistency. Additionally, protective equipment, including long sleeves, gloves, and safety glasses, was worn throughout data acquisition [20].

Calibration employed an uncontaminated sample serving as an intrinsic fluorescence blank, thereby eliminating the need for separate blank measurements. This non-destructive method allows subsequent chemical analyses of the same samples, if needed.

Data acquisition and instrument control were managed using custom LabView^®^ software (version Q3), which coordinated the laser operation, sample positioning via translation stages, monochromator settings, and CCD detection. The collected spectral data were subsequently processed using MATLAB^®^ software (version R2022A).

### 2.5. Statistical Analysis

All statistical analyses were performed using R software (version 4.1.3) within the RStudio interface. The raw fluorescence spectra were initially smoothed using a moving average filter to reduce noise. The analysis focused on the fluorescence intensities at characteristic emission wavelengths corresponding to fluoranthene (460 ± 10 nm) and pyrene (370 ± 10 nm and 395 ± 10 nm).

To normalize the data distribution and stabilize the variance across different conditions, a log_10_(x + 1) transformation was applied to all fluorescence intensity values before analysis. This approach reduces skewness and mitigates heteroscedasticity, thereby improving the reliability of non-parametric statistical tests.

Trends in fluorescence intensity across increasing PAH concentrations were evaluated using the Jonckheere-Terpstra trend test for ordered alternatives. Pairwise comparisons between the exposure groups and controls were conducted using the Kruskal–Wallis test, followed by Dunn’s post hoc multiple comparison procedure.

Homogeneity of variance assumptions were assessed with Levene’s test; due to non-normality of data distributions demonstrated by this test, non-parametric methods were preferred throughout. Statistical significance was set at α = 0.05 for all tests.

Finally, inter-individual variability within each experimental group was quantified by calculating the coefficient of variation (CV), expressed as a percentage of the mean fluorescence intensity.

## 3. Results

### 3.1. Fluorescence Spectrum Analysis

**Figure 2:**
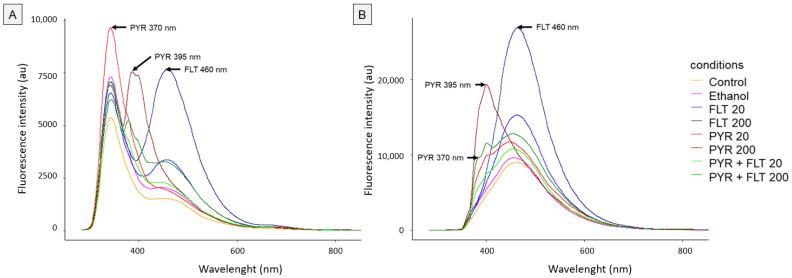
Average fluorescence emission spectra of snail viscera for each exposure modality after excitation at 266 nm (**A**) and 355 nm (**B**). Fluorescence intensity is normalized in arbitrary units (au).

We recorded average fluorescence spectra for fluoranthene and pyrene at excitation wavelengths of 266 nm and 355 nm (Figure 2). Fluoranthene exhibited a characteristic emission peak at 460 nm, consistent with Chen et al. (1994) [21], reaching intensities of approximately 7500 au at 266 nm excitation and 25,000 au at 355 nm excitation. Pyrene showed two emission peaks at 370 nm and 395 nm, following Schwarz and Wasik (1976) [22], with intensities near 9000 au and 10,000 au at 266 nm and 355 nm excitation, respectively (370 nm peak), and 7500 au and 20,000 au at 266 nm and 355 nm excitation, respectively (395 nm peak).

In contrast, control snails exposed to unspiked Helinove and Helinove spiked with ethanol exhibited low baseline fluorescence levels across all excitation and emission wavelengths compared to the PAH-exposed groups (Figure 2). Notably, the ethanol control at 370 nm with 266 nm excitation displayed fluorescence intensities overlapping and exceeding those of the other treatments (Figure 2A). Furthermore, the fluorescence intensity for PYR 20 was 1.3 times higher than PYR 200 under this condition, indicating no clear concentration-dependent response at 266 nm for pyrene analysis. Taken together, these observations suggest the limited relevance of 266 nm excitation for pyrene quantification in land snails.

### 3.2. Inter-Individual and Intra-Individual Variability

**Figure 3:**
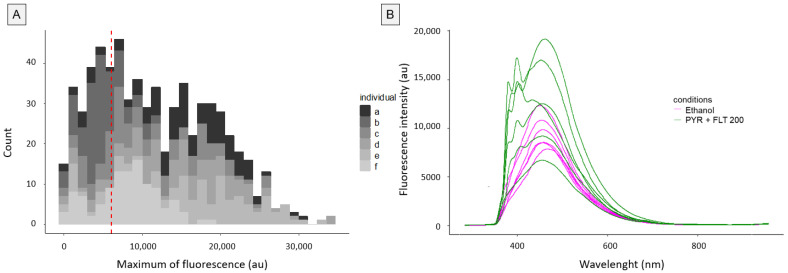
(**A**). Histogram of the 660 fluorescence maxima (110 per individual) at 395 nm after excitation at 355 nm, of the 6 individuals (a to f) of the PYR+FLT 200 condition. The vertical line represents the mean of the 6 median fluorescence maxima for each individual. (**B**). Fluorescence emission spectra of the 6 individuals in the ethanol condition and the 6 individuals in the PYR+FLT 200 condition after excitation at 355 nm. Fluorescence intensity is normalized in arbitrary units (au).

Next, we explored the variability in fluorescence signals both within and between individual snails exposed to the PYR+FLT 200 modality. A histogram of the fluorescence maxima at 395 nm following excitation at 355 nm revealed marked heterogeneity (Figure 3A). Each snail was measured 110 times, showing wide intra-individual variation; for example, three snails exhibited fluorescence signals ranging from 0 to 35,000 au, while one showed a narrower range up to 12,000 au. This high intra-individual variability underscores the importance of repetitive measurements to ensure reproducibility.

**Figure 4:**
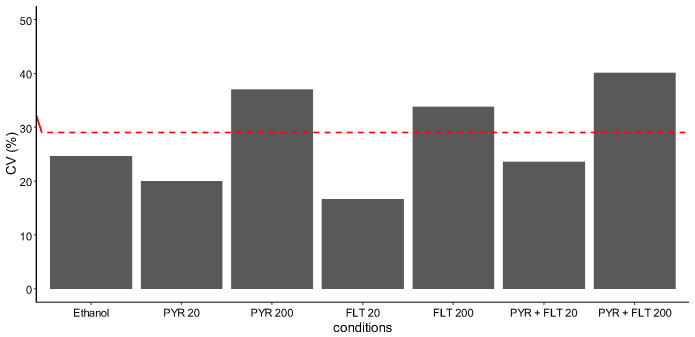
Comparison of the coefficient of variation (CV) for each condition in the pyrene peak at 395 nm, after excitation at 355 nm, as function of the red horizontal line representing the CV (%) for the control condition (Helinove without ethanol).

Moreover, inter-individual variability was substantially greater among PAH-exposed snails (CV ~40%) compared to ethanol controls (CV ~25%) (Figure 3B). Importantly, variability increased with exposure concentration by more than 15% across all conditions: PYR 20 (+19.9%), FLT 20 (+16.7%), and PYR+FLT 20 (+23.7%) rising to PYR 200 (+36.9%), FLT 200 (+33.7%), and PYR+FLT 200 (+40.2%) (Figure 4). Interestingly, such increases in variability were also observed in conditions where no fluorescence peak was expected (e.g., FLT 200 at 395 nm), suggesting other factors may contribute to signal heterogeneity. Additionally, pyrene peaks were absent in approximately 16% of individuals, further reflecting biological variability (Figure 3B).

### 3.3. Relationship Between Exposure Concentrations and Fluorescence Intensity

**Figure 5:**
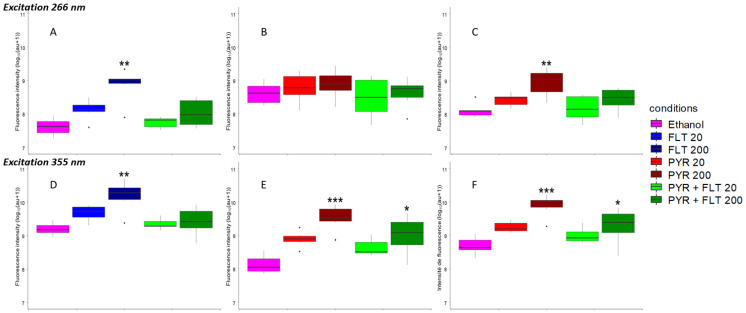
Medians of the fluorescence maxima for six individuals per experimental condition for the fluoranthene peak at 460 ± 10 nm (**A**,**D**), the pyrene peak at 370 ± 10 nm (**B**,**E**), and the pyrene peak at 395 ± 10 nm (**C**,**F**) after excitations at 266 nm and 355 nm, respectively. Boxplots represent the distribution of values: the central line corresponds to the median, the upper and lower bounds of the box indicate the first and third quartiles, whiskers extend to 1.5 times the interquartile range, and points outside this range are considered outliers. Statistical significance is indicated by asterisks (*), with * *p* < 0.05, ** *p* < 0.01, and *** *p* < 0.001. Fluorescence intensity is normalized in log10(arbitrary units (au) + 1) to facilitate comparisons between each modality and wavelength.

We then investigated the relationship between exposure concentrations and fluorescence intensity. For single exposures to fluoranthene, both concentrations elicited a statistically significant increase in fluorescence intensity at the fluoranthene peak compared to ethanol controls (*p* < 0.001), with a dose-dependent increment observed at the higher concentration (FLT200; *p* < 0.01) (Figure 5A). Similarly, pyrene single exposures showed significant increases at the 395 nm peak (*p* < 0.001), with higher concentration (PYR200) further enhancing fluorescence intensity (*p* < 0.01) (Figure 5C). However, no significant change was detected at the pyrene peak at 370 nm for either concentration (*p* = 0.11) (Figure 5B).

In contrast, mixture exposures did not significantly alter fluorescence intensities at any peaks or conditions when excited at 266 nm (*p* > 0.05) (Figure 5B). At the lower exposure concentration of 20 mg·kg^−1^, although the median fluorescence appeared elevated for single exposures at respective peaks compared to controls, these differences did not reach statistical significance.

When excited at 355 nm, similar patterns emerged: fluoranthene single exposure induced significant fluorescence increases at both concentrations (*p* < 0.001), with a greater effect for FLT200 (*p* < 0.01) (Figure 5D). Pyrene single exposures significantly elevated fluorescence at both peaks (*p* < 0.001), with higher concentrations producing larger effects (*p* < 0.01) (Figure 5E,F). Unlike the results at 266 nm excitation, mixture exposures showed significant increases in pyrene peaks (*p* < 0.005; highest concentration *p* < 0.05), although the fluoranthene peak intensity did not significantly increase (*p* = 0.072). At lower doses, increases remained statistically non-significant.

Despite some non-significant findings, many experimental conditions exhibited clear dose-dependent trends in median fluorescence values, supported by Jonckheere-Terpstra trend tests (*p* < 0.001). This overall pattern reflects a consistent positive correlation between the exposure concentration and fluorescence response across single and mixture exposures.

## 4. Discussion

### 4.1. Inter-Individual Variability of PAH Bioaccumulation

This study highlights a substantial inter-individual variability in PAH bioaccumulation among land snails exposed to the same contaminant concentrations, underscoring the sensitivity and utility of UV-LIF/SPS technology for environmental risk assessment (ERA). Such variability aligns with ecophysiological expectations and has been documented previously in snails exposed to moderately contaminated soils containing the 16 priority PAHs. For instance, Morin-Crini et al. (2020) observed similar patterns after a 28-day ex situ exposure of *C. aspersus* sub-adult snails to soil contaminated with 139.6 mg·kg^−1^ total PAHs, where pyrene and fluoranthene were predominant (25.7 mg·kg^−1^ and 36.6 mg·kg^−1^, respectively) [23]. Comparable inter-individual variations have also been reported in marine molluscs such as Buccinidae whelks *Neptunea lyrata,* and *Buccinum undatum,* following dietary exposure to 1-hydroxy-pyrene [24,25].

Several factors may explain these individual differences. Feeding behavior influences internal PAH concentrations [23], while the strong affinity of PAHs for lipids suggests that normalizing concentrations to tissue lipid content could mitigate variability [26], as demonstrated by Ferguson and Chandler (1998) for the estuarine polychaete *Streblospio benedicti* [27]. Furthermore, reproductive and spawning status can cause significant fluctuations in both lipid and PAH levels [28,29,30]. The observed variability is likely due to our focus on adult reproductive individuals. Additionally, genetic or physiological differences affecting absorption, distribution, metabolism, and excretion, particularly the activities of cytochrome P450 and glutathione-related enzymes, may further influence bioaccumulation outcomes [31]. This hypothesis is supported by the literature and should be confirmed in future studies.

Recognizing these inter-individual differences is crucial for ecotoxicological risk assessments, as it enables estimation of the fraction of the population at highest risk. This, in turn, refines risk characterization and uncertainty quantification, especially in site-specific ERA frameworks such as TRIAD [4]. However, data gaps remain regarding PAH bioaccumulation and metabolism in land snails. Our findings emphasize the importance of analyzing multiple individuals rather than relying on single samples to obtain reliable and ecologically representative insights into contaminant transfer within populations.

### 4.2. Application in Environmental Risk Assessment

A notable advantage of the semi-targeted UV-LIF/SPS approach is its potential to detect not only parent PAHs but also structurally related compounds. This includes early biotransformation products, as suggested by Ismert et al. (2002) in their studies on naphthalene metabolism in snails [31]. Although the fluorescence behavior of PAH metabolites in land snail digestive glands remains poorly understood, analogous fluorescence techniques have successfully identified 1-hydroxy-pyrene in marine polychaetes exposed to pyrene-contaminated sediments [32].

Given that PAHs often occur as complex mixtures in contaminated soils [33], the capacity of a single UV-LIF measurement to simultaneously identify pyrene and fluoranthene strengthens the prospects for this technology’s application in ERA using bioindicator species. Moreover, characterizing inter-individual variability in internal PAH concentrations allows refinement of risk assessments by incorporating the proportion of highly affected organisms.

Importantly, our method is non-destructive to biological materials; for example, the same digestive gland sample can subsequently be used for biomarker analyses linking environmental PAH bioavailability with organismal responses. This dual use enhances the WoE approach in ERA and has been demonstrated in studies correlating metal(loid) exposure with sexual maturation inhibition and genotoxicity in snails [34,35]. By enabling simultaneous chemical and biological analyses of individual organisms, this approach reduces the number of individuals required for ecotoxicological studies and addresses inter-individual variability more effectively.

From an environmental perspective, UV-LIF/SPS offers a greener alternative to conventional analytical methods such as QUEChERS extraction followed by GC-MS/MS quantification—the current gold standard for PAH analysis in organisms like snails [19]. Our technique eliminates reagent consumption, organic solvents, gases, and plastic microtubes, and generates minimal chemical waste, limited to reusable biological material. These characteristics align well with the principles of green and white analytical chemistry, which emphasize the synergy between analytical performance, environmental sustainability, and practical feasibility [10,36,37].

Finally, our findings demonstrate that UV-LIF/SPS enables high-throughput analysis (<4 min per sample), which is markedly faster than traditional GC-MS/MS workflows that require several hours for extraction, purification, and quantification [23]. This efficiency advantage positions UV-LIF/SPS as a promising tool for rapid screening in environmental monitoring programs.

### 4.3. Limitations of the Study

While this study demonstrates the potential of UV-LIF combined with solid-phase spectroscopy for detecting PAHs in land snails, certain aspects warrant further investigation to strengthen the method’s applicability. The complex biological matrix of snail tissues may contain naturally fluorescent compounds that could partially overlap with PAH signals. In particular, endogenous molecules such as porphyrins, flavins, or lipofuscin derivatives, which are naturally present in molluscan tissues, can emit in similar spectral regions, potentially leading to background noise or signal confounding. Although the characteristic excitation/emission maxima of pyrene and fluoranthene used in this study reduce this risk, the possibility of partial spectral overlap remains, especially in samples with high endogenous fluorescence. Although no specific assignment of endogenous fluorophores was attempted, control individuals were analyzed under the same conditions, providing a relevant spectral baseline. This allowed us to distinguish the background signals from pollutant-induced features with reasonable confidence. Nonetheless, future studies should further investigate the nature and variability of tissue autofluorescence to enhance the spectral resolution. In addition, future studies should include the spectral characterization of potential interferents in snail tissues to better discriminate background signals from pollutant-specific peaks. However, the distinct fluorescence peaks of pyrene and fluoranthene used in this study helped mitigate this issue. To improve the specificity and interpretability of complex fluorescence data, this could be complemented by the use of chemometric approaches or spectral subtraction techniques. In particular, tools such as Principal Component Analysis (PCA) and Partial Least Squares Discriminant Analysis (PLS-DA) may help reduce data dimensionality, reveal spectral patterns associated with contaminant exposure, and improve the classification of exposed versus control individuals [38,39,40]. These multivariate approaches are increasingly relevant in ecotoxicology for interpreting subtle biochemical variations in solid-phase fluorescence spectra from biological matrices. Extending this method to a broader range of PAHs and their metabolites is essential to fully establish its scope and reliability. Overall, this study was limited to two exposure concentrations for fluoranthene and pyrene. To fully assess the utility and quantitative performance of the UV-LIF/SPS method, future research should include detailed dose-response experiments across a broader range of concentrations and contaminants. A lipid normalization could be explored in future optimization studies to improve quantification due to the interaction of PAHs with lipids, as demonstrated by Shin et al. 2025 with human data from the Korean National Environmental Health Survey [41]. Although this technique offers rapid and non-destructive analysis, further validation through direct comparison with established methods such as GC-MS/MS is needed to confirm its sensitivity and accuracy across different sample types. This preliminary study employed controlled laboratory exposures using spiked diets. Future research should assess the performance of this technique using naturally contaminated samples under field conditions to confirm its real-world applicability. Such validation will be essential for evaluating the robustness and practical utility of the developed method in field settings. Finally, the observed inter-individual variability reflects ecological and physiological differences among snails and highlights the importance of adequate replication for robust assessments.

### 4.4. Future Milestones and Perspectives

This study establishes a strong foundation for applying UV-LIF combined with solid-phase spectroscopy (SPS) to detect PAHs in land snails. However, several important next steps must be undertaken to advance this method toward routine environmental monitoring and risk assessment.

First and foremost, comparative analyses involving UV-LIF/SPS and the established gold standard GC-MS/MS technique are essential [23]. Such comparisons will enable calibration of fluorescence spectra against absolute PAH concentrations, thereby allowing accurate quantification and validation of the fluorescence-based approach across various environmental samples. To support this effort, preparing spiked standard samples containing both individual PAHs and complex mixtures will be critical for thoroughly evaluating the method’s accuracy, precision, linearity, and reproducibility.

In addition to experimental validation, developing advanced chemometric tools or artificial intelligence algorithms with machine learning is highly recommended. These computational approaches will enhance signal deconvolution and compound identification, particularly in samples containing multiple co-occurring PAHs, ultimately improving data interpretation and facilitating application in complex environmental matrices. In this first proof-of-concept study aiming to demonstrate the feasibility of UV-LIF/SPS for PAH detection in a biological matrix, it is not yet possible to definitively position this technology within the analytical hierarchy alongside the GC-MS/MS approach.

Moreover, expanding the scope of the method to include the detection of a wide range of PAHs and their metabolites, notably the 16 priority PAHs identified by the US EPA, as well as hydroxylated PAH metabolites (OH-PAHs), will provide deeper insights into biotransformation processes and organism exposure profiles. This extension is supported by analogous studies on other organisms [42,43]. Complementing this, integrating non-lethal sampling techniques, such as hemolymph analysis, which can be performed without sacrificing snails [35], offers a promising means for longitudinal monitoring of PAH bioavailability and toxicity while minimizing impacts on test populations.

Furthermore, field validation studies are imperative to assess the robustness, scalability, and applicability of UV-LIF/SPS under naturally contaminated conditions. These investigations will be pivotal in demonstrating the readiness of the technique for implementation in regulatory environmental monitoring programs.

Finally, combining UV-LIF/SPS analyses with biomarker assays on the same biological samples will strengthen ecological risk assessments by directly linking chemical exposure to biological effects within individual organisms [34,35]. This integrated strategy supports more ethical and efficient testing by reducing the number of animals required and improving the overall WoE used for environmental decision-making.

These milestones and perspectives collectively outline a clear and coherent path toward establishing UV-LIF/SPS as a rapid, sensitive, non-destructive, and environmentally friendly tool for high-throughput monitoring of PAHs in terrestrial bioindicators. Ultimately, this advancement promises to enhance the precision and sustainability of ERA practices.

## 5. Conclusions

This study presents a novel UV-LIF/SPS platform for rapid detection of PAH transfer to land snails using pyrene and fluoranthene as model compounds. Our method offers the following advantages: (i) high-throughput analysis in under 4 min per sample, (ii) non-destructive measurement preserving biological matrices for further chemical or biomarker analyses, (iii) clear fluorescence signals correlated with exposure concentrations, (iv) applicability to single and mixed PAH exposures, (v) ability to capture inter-individual variability critical for ecological risk assessment, (vi) alignment with green analytical chemistry principles by minimizing reagent use and waste, (vii) minimal sample preparation, and (viii) adaptability for extension to other PAHs and environmental matrices. Due to its simplicity, speed, and eco-friendliness, this platform has strong potential for routine environmental monitoring of PAH bioavailability using bioindicator species. Future work will focus on validating the method against conventional techniques (e.g., GC-MS/MS), establishing dose-response models for multiple PAHs, and testing the approach under field conditions. These steps are critical for supporting the integration of UV-LIF/SPS into environmental risk assessment (ERA) frameworks.

## Figures and Tables

**Table 1:** Exposure conditions.

Condition Names	Spiked Concentrations by the Dosed Addition Method (mg·kg^−1^ Dry Weight)	PAH Spiked	Ethanol Used for Spiking	Number of Replicates (Faunarium)	Number of Snails per Replicate (Faunarium)
**Control**	0	No	No	3	6
**Ethanol**	0	No	Yes	3	6
**PYR20**	20	PYR	Yes	3	6
**PYR200**	200	PYR	Yes	3	6
**FLT20**	20	FLT	Yes	3	6
**FLT200**	200	FLT	Yes	3	6
**PYR+FLT20**	20	PYR+FLT	Yes	3	6
**PYR+FLT200**	200	PYR+FLT	Yes	3	6

## Data Availability

Data are available upon request to the corresponding author.
